# Bilateral Life-Threatening Obstructive Cystine Stones in a 19-Month-Old Child Requiring Staged Endourological Management: A Case Report

**DOI:** 10.7759/cureus.104152

**Published:** 2026-02-23

**Authors:** Ezat Bakhsh, Mohamed A Ahmadi, Ahmed A Al Rashed, Husain Jaafar, Omran Hasan, Qasim M Isa, Arka Chatterjee, Sayed Hasan Ebrahim, Basma D Malalla, Abdolsalam Ahmadi, Nader Awad

**Affiliations:** 1 Pediatric Surgery, Salmaniya Medical Complex, Manama, BHR; 2 Urology, Salmaniya Medical Complex, Manama, BHR

**Keywords:** acute kidney injury care, cystine stones, cystinuria, pediatric urology surgery, percutaneous nephrolithotomy (pcnl)

## Abstract

Cystinuria is an inherited disorder that can lead to recurrent stone disease with a very early onset and possible devastating effects. Severe presentations in infancy are uncommon but may be life-threatening, particularly when obstruction is bilateral, which can cause acute renal injury and impairment. We report a 22‑month‑old boy who presented with anuric acute kidney injury, severe hyperkalemia, and ventricular tachycardia secondary to bilateral obstructing cystine stones. He initially required emergency decompression, followed by staged percutaneous nephrolithotomy (PCNL) procedures aided by retrograde intrarenal procedures. This case highlights the need for rapid decompression, careful staging of definitive surgery, and long‑term metabolic management in young children with cystinuria.

## Introduction

Cystinuria is one of the most prevalent inherited causes of nephrolithiasis within the pediatric population, arising from defective proximal tubular reabsorption of cystine and dibasic amino acids [1]. The pathophysiology occurs due to mutations in the SLC3A1 or SLC7A9 genes, which lead to an increased concentration of the already intrinsically poorly soluble cystine in urine and hence predispose affected individuals to recurrent stone formation from an early age [1]. This is why early-onset nephrolithiasis in infants and toddlers should raise a strong suspicion for an underlying metabolic or genetic disorder.

In this developmental stage, clinical presentations are often nonspecific, with symptoms such as reduced feeding, lethargy, or vomiting frequently leading to delayed recognition until substantial obstruction or renal dysfunction occurs [1]. The subtle clinical signs may lead to misdiagnosis or delayed intervention, which can have profound consequences on the child's renal health. Therefore, it is crucial for healthcare providers to maintain a high index of suspicion for cystinuria in children presenting with recurrent or atypical urinary symptoms [2]. Specific urinary tests, including amino acid analysis and urine pH measurements, can facilitate timely diagnosis by demonstrating elevated levels of cystine and low urine pH levels and hence warranting further evaluation [2]. Management of cystinuria is typically multifaceted, encompassing both dietary modifications and pharmacologic interventions aimed at reducing cystine concentration in urine; however, even with optimum medical management, surgical intervention may still be required, especially in cases of large or obstructive stones [2].

Moreover, cases of bilateral stone disease in young children adds a set of unique diagnostic and therapeutic challenges. Factors such as limited physiological reserve, smaller anatomical landmarks, and restricted surgical space demand meticulous planning and a multidisciplinary approach to management. Pediatric cystine stones often present as large, bilateral calculi that are resistant to conventional therapies like shock wave lithotripsy, frequently necessitating more invasive endo-urological interventions [3]. While typical presentations may include pain, hematuria, or urinary tract infection, the acute onset of bilateral obstruction with significant metabolic complications is a rare occurrence [3].

In this report, we describe a case of a toddler with bilateral cystine stones that led to life-threatening complications which were managed through staged endo-urological procedures. We believe that such cases enhance awareness of infrequent but severe clinical scenarios and also facilitate the sharing of effective management strategies, which may serve as vital resources for pediatric centers facing similar challenges.

## Case presentation

A 19‑month‑old boy who did not have any previous medical history presented in July 2025 with nonspecific symptoms of reduced activity, vomiting, and markedly decreased urine output, as reported by his parents, for a duration of one day. Upon arrival at the emergency department (ED), his physical examination showed a lethargic infant with decreased responsiveness associated with anuria. However, his abdomen was soft and lax, with no evidence of suprapubic dullness or fullness, and his vitals are summarized in Table 1.

**Table 1:** Vital signs and weight at emergency department presentation

Vitals	Reading	Unit
Heart rate	124	Beats per minute (BPM)
Blood pressure	114/76	mmHg
Temperature	36.4	Celsius
Respiratory rate	45	Breaths per minute
Weight	11.9	Kilograms (Kg)

His initial blood panel demonstrated severe metabolic derangements consistent with acute kidney injury (AKI), including a serum creatinine level of 735 µmol/L and severe hyperkalemia (10 mmol/L). Pertinent laboratory findings are summarized in Table 2.

**Table 2:** Lab values at emergency department presentation

Reading	Values	Unit	Reference range
White cell count	7.46	x 10^9^/L	6.0-13.5
Hemoglobin	9.3	g/dL	10.1-12.05
Creatinine	735.0	µmol/L	11.0-36.0
Urea	40.2	mmol/L	1.8-6.4
Potassium	10	mmol/L	3.5-5.1
Sodium	130	mmol/L	136-145
Bicarbonate	15	mmol/L	20-31

While he was still in the ED, he developed an episode of ventricular tachycardia due to underlying electrolyte and metabolic derangements and required synchronized cardioversion. The patient was treated as an acute life-saving presentation, and the pediatric nephrology, pediatric intensive care unit (PICU), and pediatric urology teams were involved. He was admitted to the PICU and required hemodialysis under the supervision of the pediatric nephrology and PICU teams, with a blood flow of 3-5 mL/kg/min, including potassium-free dialysate with minimal ultrafiltration. This was performed under cardiac monitoring and followed by strict input-output monitoring and hydration, after which renal function and electrolyte derangements improved (creatinine returned to 81 µmol/L and potassium to 3.4 mmol/L), at which point he was stable enough to be taken for surgery.

**Figure 1:**
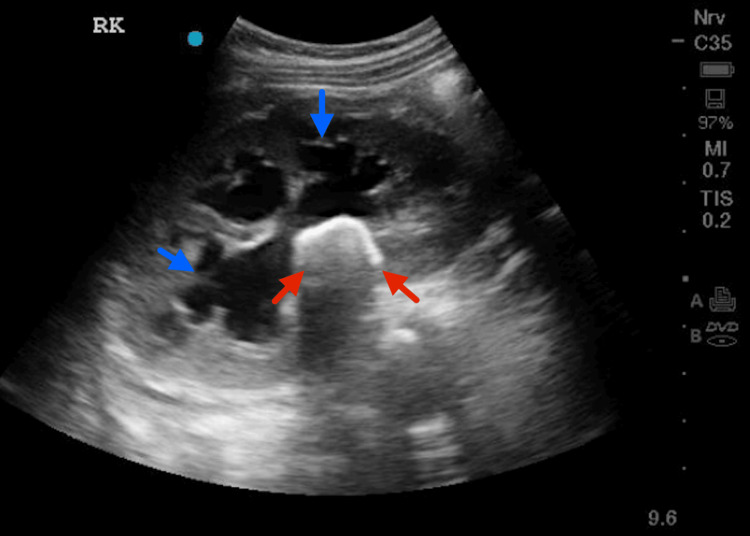
Ultrasound of the right kidney demonstrating renal calculi with hydronephrosis The blue arrows point to the dilated renal calyxes, and the red arrows point toward the renal calculi with distal hypoechoic shadows.

**Figure 2:**
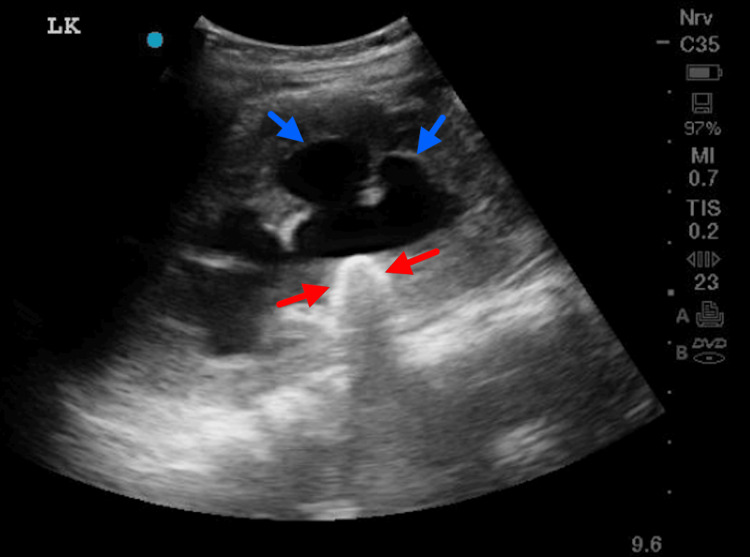
Ultrasound of the left kidney demonstrating renal calculi with hydronephrosis The blue arrows point to the dilated renal calyxes, and the red arrows point to the renal calculi with distal hypechoic shadows.

He also initially underwent a plain abdominal X-ray, which showed faint radiopaque shadows within both kidneys, consistent with bilateral staghorn renal calculi. This was followed by an urgent renal ultrasound, which showed bilateral hydronephrosis with renal calculi and preserved corticomedullary differentiation, as demonstrated in Figures 1, 2.

**Figure 3:**
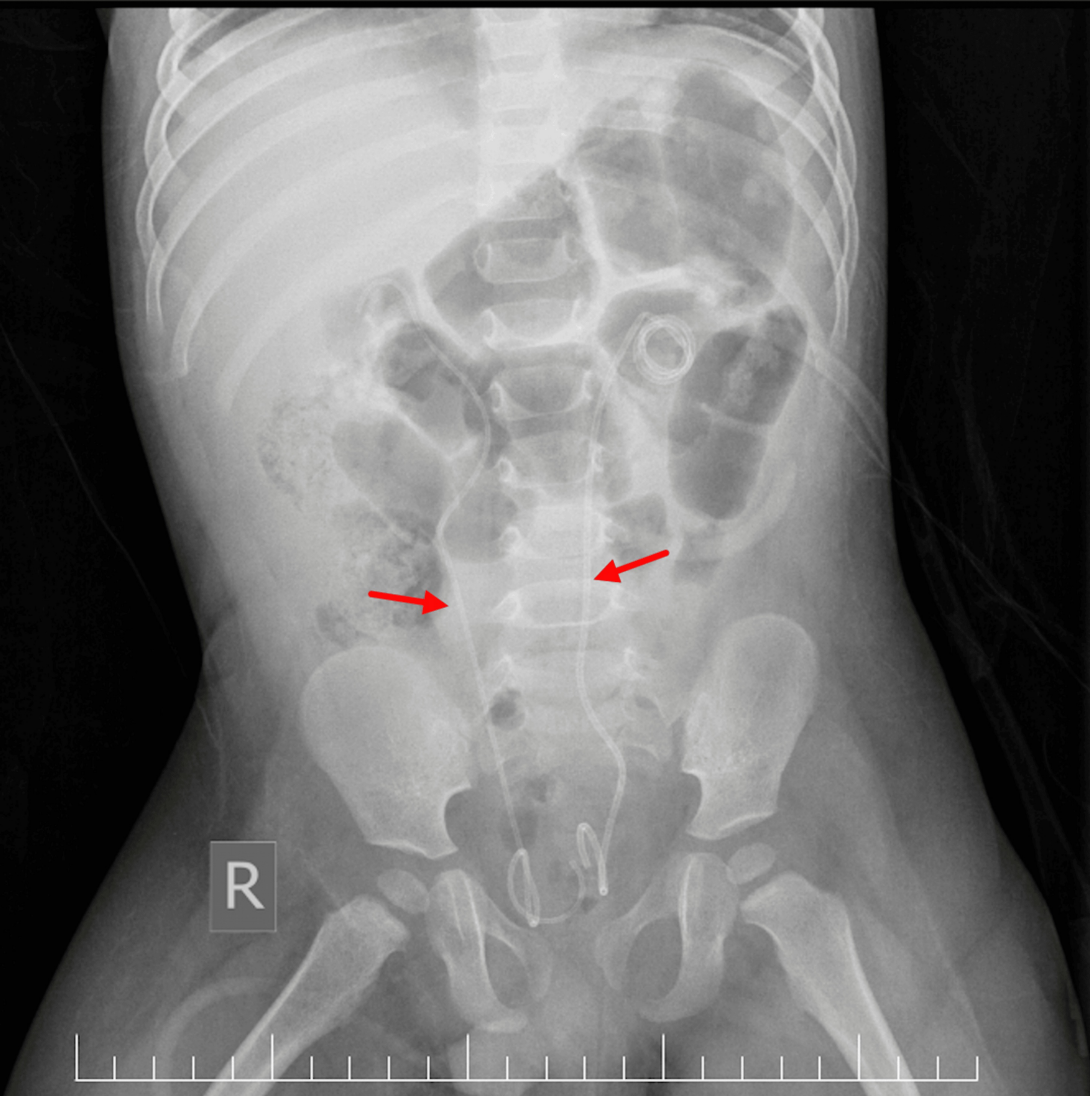
Post-cystoscopy abdominal X-ray Bilateral double-J (DJ) stents in place, demarcated by red arrows.

Following these findings, the patient underwent emergency cystoscopy and retrograde pyelography, which confirmed bilateral pelvi-ureteric junction obstruction due to stones, and bilateral Coloplast 4.7 Fr, 18 cm double-J (DJ) ureteric stents were inserted, as demonstrated in Figure 3.

**Figure 4:**
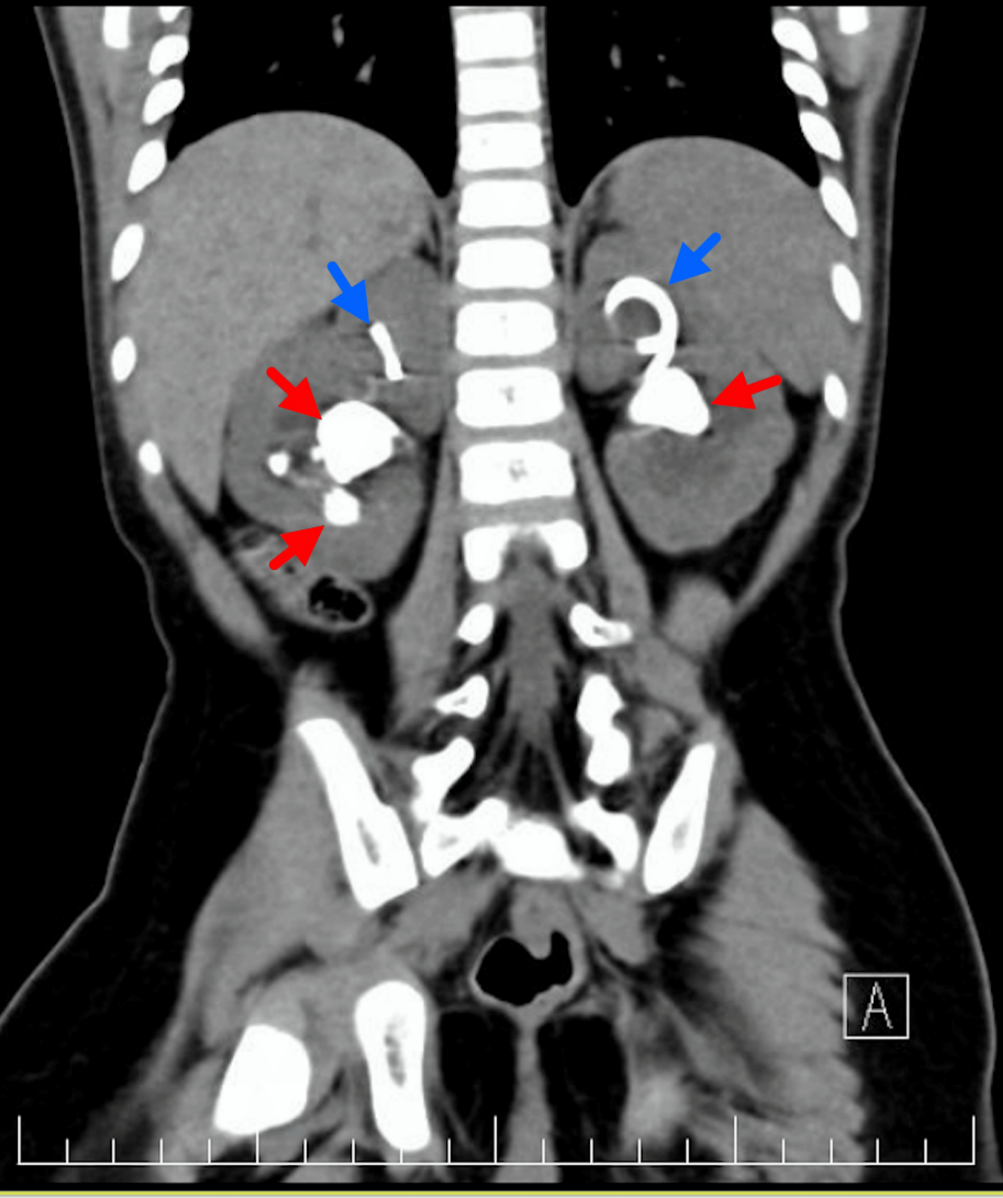
Coronal view of non-contrast-enhanced CT of the abdomen and pelvis Bilateral staghorn calculi are indicated by the red arrows, and the renal tips of the double-J (DJ) stents are indicated by blue arrows.

**Figure 5:**
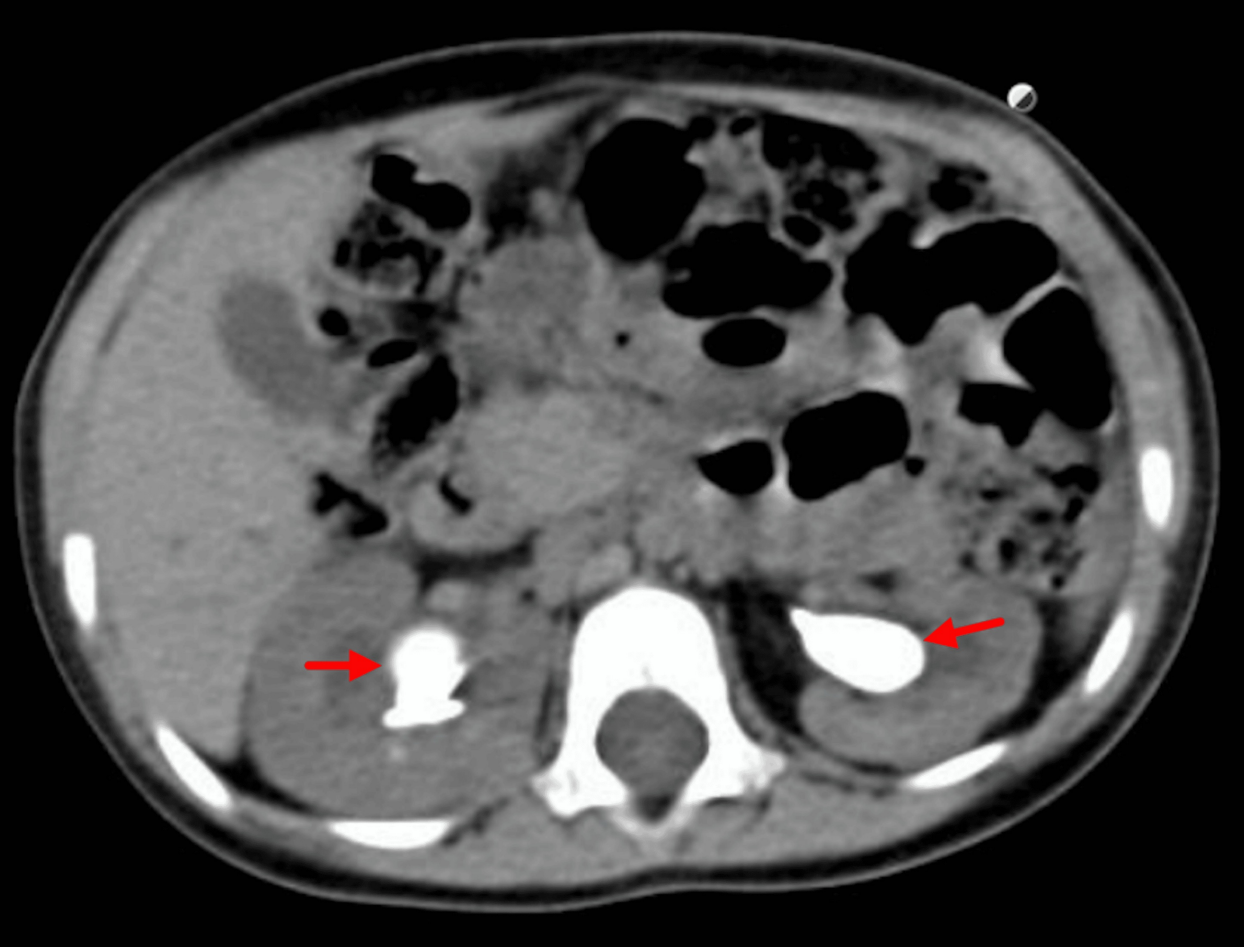
Axial view of non-contrast-enhanced CT of the abdomen and pelvis Bilateral staghorn calculi are indicated by the red arrows.

The patient continued to be managed in the PICU post bilateral DJ insertion, with gradual improvement in his urine output. He also developed post‑obstructive diuresis, hypernatremia, and reactive hypertension, all of which resolved with supportive management. Furthermore, he also received one unit of packed red blood cell transfusion for anemia and was treated with low‑molecular‑weight heparin for a femoral line-associated deep vein thrombosis.Once the patient was stabilized, he was shifted out of the PICU into the regular pediatric ward and then discharged home. Definitive management of the stone was scheduled on an elective basis, and non-contrast-enhanced CT scan of the abdomen and pelvis was arranged, with findings shown in Figures 4, 5.

At 21 months of age, he was electively admitted for definitive stone management. He underwent cystoscopy, right DJ stent exchange, and left percutaneous nephrolithotripsy (PCNL). Percutaneous access was obtained under fluoroscopic and ultrasound guidance with interventional radiology support, and this was followed by progressive tract dilatation and insertion of a 16 Fr Amplatz sheath. Through this, holmium laser (Karl Storz ENDOSKOPE laser) lithotripsy of the stones was undertaken using a 365-µm fiber until the renal pelvis and calyces were cleared of all stones. A 16 Fr Malecot nephrostomy tube and a 4.7 Fr, 18 cm DJ stent were left in situ, and stone fragments were sent for analysis. Stone analysis demonstrated cystine stones (80%) with a potassium urate component (20%). Genetic testing for cystinuria-related mutations was initiated.

**Figure 6:**
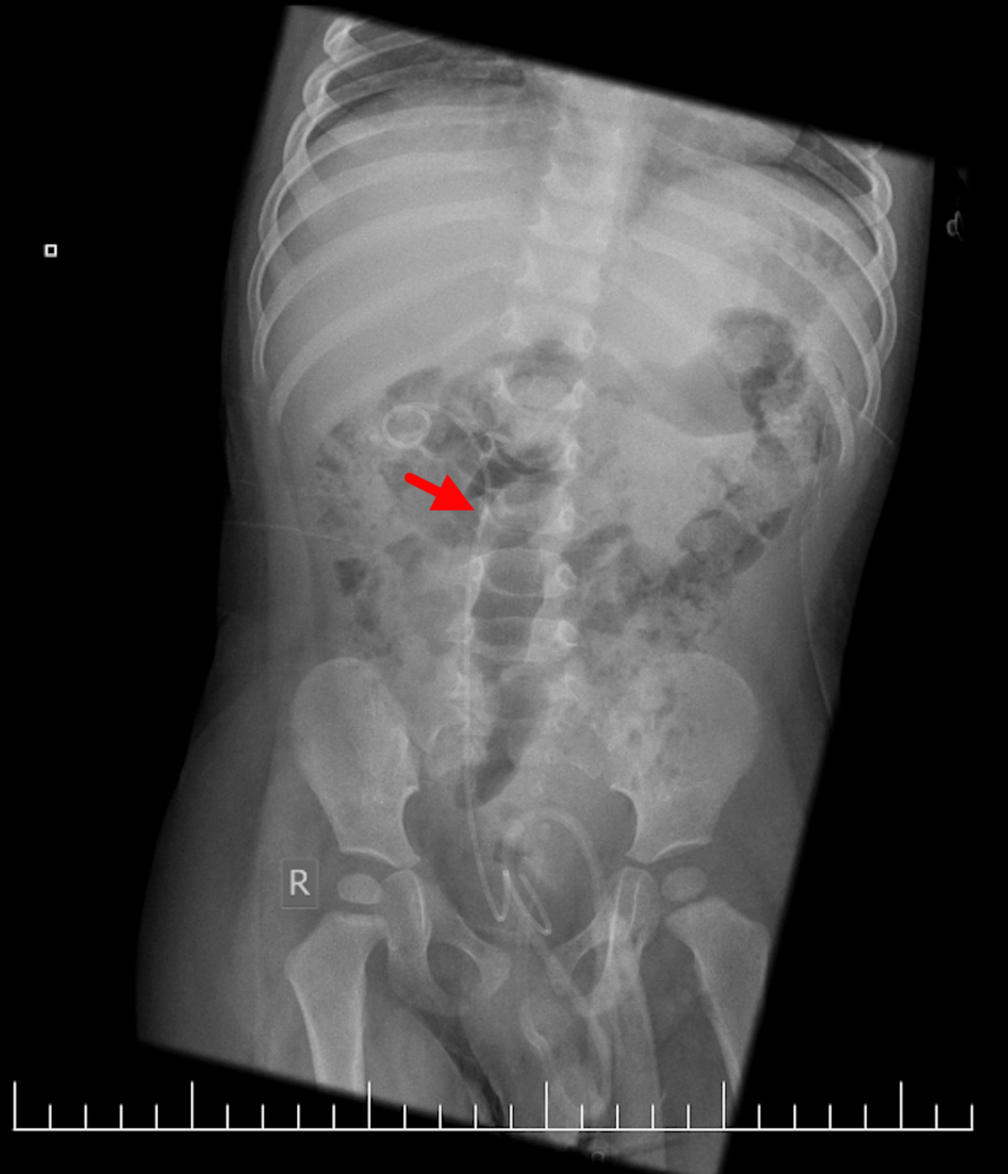
Post-right PCNL Right DJ stent in situ (red arrow), with no large residual stone fragments noted. PCNL: Percutaneous nephrolithotomy; DJ: Double-J.

After approximately one month, at 22 months of age, he underwent a planned right-sided PCNL for persistent stone burden on the right side. Intraoperatively, a large pelvic stone with multiple fragments was identified, and lithotripsy was undertaken in a similar fashion to the first session, with similar operative steps as mentioned above for the left side. Near-complete lithotripsy was achieved, with tiny fragments remixing. A right 4.7 Fr, 18 cm DJ stent was left in place, and the left DJ stent was removed. The postoperative course was uncomplicated, and renal function remained stable. The patient currently has complete clearance of all stones, and following the second PCNL session, a single DJ stent was seen on the right side (Figure 6).

The patient was discharged in stable condition and is currently following up with pediatric nephrology for medical management of cystinuria and with the urology team for postoperative care, removal of the right DJ stent, and follow-up. His renal function has returned to within normal ranges, and he currently has good urine output; however, he will need lifelong surveillance for stone recurrence.

## Discussion

This case illustrates an extreme early presentation of cystinuria with bilateral obstruction and life-threatening metabolic complications. Prompt recognition and urgent decompression were critical in reversing renal failure and stabilizing the patient. Bilateral ureteric stenting remains an effective emergency strategy in such scenarios, with delayed definitive management once the patient stabilizes. Definitive management of cystine stones in young children is challenging, as these stones are typically large, hard, and poorly responsive to shock wave lithotripsy [3]. PCNL offers the highest stone-free rates and can be performed safely in infants and toddlers in experienced centers [3].

From a surgical standpoint, achieving adequate stone clearance while limiting renal trauma is paramount. The use of staged PCNL allowed a meaningful reduction of stone burden while minimizing operative risk. Advances in miniaturized instruments and image-guided access have made PCNL increasingly safe in young children; however, such procedures should be undertaken in experienced centers with appropriate multidisciplinary support.

The evidence for management approach and outcomes in such cases spans multiple studies with consistent findings. A study conducted by Nouralizadeh et al. in 2009 showed a 79.16% stone clearance in children under 5 years of age, increasing to 91.67% with additional treatments, with stone-free status described as stones less than 3 mm on postoperative imaging [4]. Furthermore, a paper published by Yan et al. in 2012 demonstrated that surgeons achieved 85.2% complete stone clearance using mini-PCNL, rising to 92.6% with adjunctive treatments, with stone-free rates described as stones less than 4 mm on postoperative imaging [5]. Additionally, these findings were corroborated by Jones et al. in 2017, who systematically reviewed miniaturized PCNL techniques, finding stone-free rates between 80% and 100%, with relatively low complication rates of 11.2% [6].

The technical evolution of PCNL in young children has progressed from adult-sized instruments to increasingly miniaturized approaches to improve surgical and postoperative outcomes. This was first published by Jackman et al. in 1998, who pioneered the "mini-perc" technique using an 11 F peel-away vascular access sheath, demonstrating successful outcomes in children aged two to six years with minimal blood loss (average 25 mL) and no transfusion requirements [7]. This innovation led to further miniaturization, with Hosseini et al. demonstrating ultra-mini-PCNL using 12 F tracts and semi-rigid ureteroscopes, achieving a 97% stone-free rate. However, Jones et al. noted that tract size influences complication patterns, with smaller tracts associated with different risk profiles compared to standard approaches [1,3].

Even complex stone burdens in very young children can be successfully managed with PCNL techniques. Manohar et al. reported in 2006 outcomes in children aged 11 months to 4.5 years with staghorn and complex caliceal calculi, achieving an 86% stone-free rate with mean hospital stays of 3.5 days [8]. Moreover, Aron et al. specifically studied complete staghorn calculi in preschool children, demonstrating 89% complete clearance with PCNL monotherapy, increasing to 94.7% with adjunctive treatments [9].

Our case aligns well with several published experiences. The staged approach that we used matches the recommendation for staging procedures, particularly when there is a risk of operative time exceeding 60 minutes or when significant bleeding occurs. However, several aspects of our case are unique compared to the literature. Our patient's presentation with life-threatening AKI requiring cardioversion and hemodialysis represents a more severe initial presentation than typically reported. The use of laser lithotripsy contrasts with most studies that used pneumatic lithotripsy, including those by Hosseini et al. (2021) [1], Nouralizadeh et al. (2009) [4], and Yan et al. (2012) [5], all of which employed pneumatic fragmentation. The bilateral presentation with complete anuria is more severe than most reported cases as apparent in the study by Dogan et al., which studied 51 renal units but did not report cases requiring emergency dialysis [10].

The main learning point from this case report is that young age should not act as a deterrent to undertaking PCNL; however, staged procedures can optimize patient safety, particularly when patients present with AKI and severe symptoms with metabolic derangements. This case also shows, in keeping with the literature mentioned above, that staged procedures do not compromise overall outcomes while prioritizing patient safety.

## Conclusions

Cystinuria can present early in life with recurrent urolithiasis requiring lifelong surveillance. Although the disease and stone management have been described in previous literature, this case highlights a truly devastating and potentially life-threatening rare presentation of AKI, severe metabolic derangements, and the sequelae that can follow bilateral obstruction. Emergency decompression followed by staged PCNL allowed safe and effective management in this toddler. The approach to such cases should be done with the help of a multidisciplinary team, and early diagnosis and long-term metabolic management are crucial to prevent recurrence and protect renal function.
